# Associations of Dietary Diversity Trajectories with Frailty among Chinese Older Adults: A Latent Class Trajectory Analysis Based on a CLHLS Cohort

**DOI:** 10.3390/nu16101445

**Published:** 2024-05-10

**Authors:** Chenyu Zhao, Yuping Wang, Xiaocan Jia, Jingwen Fan, Nana Wang, Yongli Yang, Xuezhong Shi

**Affiliations:** Department of Epidemiology and Biostatistics, College of Public Health, Zhengzhou University, Zhengzhou 450001, China; zcy8715@gs.zzu.edu.cn (C.Z.); 202011271030324@gs.zzu.edu.cn (Y.W.); jxc@zzu.edu.cn (X.J.); fanjingwen126@126.com (J.F.); wnn0924@126.com (N.W.)

**Keywords:** Chinese older adults, frailty, dietary variety score, latent class trajectory modeling

## Abstract

Background: High dietary diversity has been found to be associated with frailty. However, the trajectory of dietary diversity intake in relation to frailty is unclear. Methods: Using the latent class trajectory modeling approach, we identified distinctive dietary variety trajectory groups among 2017 participants based on the Chinese Longitudinal Healthy Longevity Survey acquired at four time points within a 10-year period. Frailty status was assessed using a frailty index comprising 37 health deficits. Dietary diversity was quantified using the dietary variety score (DVS), based on food category consumption frequency. Logistic regression analyses were employed to explore the association between DVS change trajectories and frailty. Results: This study identified two distinct DVS trajectories: “Moderate-Slow decline-Slow growth”, encompassing 810 (40.16%) individuals, and “Moderate-Slow growth-Accelerated decline”, including 1207 (59.84%) individuals. After adjusting for covariates, the odds ratio for DVS in the “Moderate-Slow decline-Slow growth” group was 1.326 (95% confidence interval: 1.075–1.636) compared to the “Moderate-Slow growth-Accelerated decline” group. The “Moderate-Slow decline-Slow growth” trajectory continued to decrease and was maintained at a low level in the early stages of aging. Conclusion: Sustaining a high dietary diversity trajectory over time, particularly in the early stages of aging, could potentially decrease the risk of frailty among older Chinese adults.

## 1. Introduction

Frailty is a condition characterized by a decline in the functioning of multiple body systems, rendering the body more susceptible to the negative effects of stressors [1]. The prevalence of frailty varies with age. Globally, among individuals aged 60 to 69, the prevalence is 23%. In the 70 to 79 age group, it is 25%, while among those aged 80 to 89, it reaches 32%. For individuals aged 90 and above, the prevalence of frailty is notably higher, at 61% [2]. Meanwhile, frailty is strongly linked to unfavorable health outcomes and a notable rise in healthcare expenditures [3,4]. The population with frailty has been associated with increased risks of aging-related diseases, such as sarcopenia [5], polypharmacy [6], and ischemic heart disease [7], which seriously affect the health of older people. The global impact of frailty is expected to increase due to an increasingly aging population. Consequently, the identification of risk factors and the proactive addressing of frailty represent pressing public health imperatives [8].

Unlike the natural aging process, frailty is a condition that can be mitigated or even reversed through suitable interventions and preventive measures. In addition to the demographic characteristics that are associated with frailty, addressing poor lifestyle choices and mitigating the effects of childhood adversity may help reduce the risk of frailty [9,10,11]. In recent years, many researchers have focused on the effects of dietary diversity on frailty. Previous studies have indicated that dietary diversity has the potential to improve the frailty status of older people. Three cross-sectional studies suggest that low dietary variety is associated with frailty in older people and contributes to a higher prevalence of frailty [12,13,14]. Particularly among older persons who live alone, there is a notable trend toward higher frailty scores and lower dietary diversity [15]. Findings from two cohort studies indicate a heightened need for dietary support to reduce the onset of frailty in older adults, with a particular emphasis on older women [16,17]. At the same time, studies focusing on older adults in China suggest that a high food variety in one’s diet may contribute to a lower incidence of frailty [18,19]. Since dietary diversity is a dynamic and heterogeneous process, it may be better captured by repeatedly measuring the intake frequency of multiple foods over time. However, most studies have only collected information on dietary diversity at a single time, and few studies have examined the longitudinal pattern of dietary diversity as well as its associations with frailty. Latent class trajectory modeling (LCTM) offers a valuable approach to categorizing heterogeneous groups into more homogeneous ones while still allowing for discerning the distinctions between individuals and groups [20]. This modeling technique is commonly used to depict the trajectory of a quantitative variable over time [21]. Remarkably, there is limited research exploring the association between dietary diversity trajectories and frailty in older Chinese adults.

To enhance the quality of life during later years, older individuals should prioritize reducing the risk of frailty. This study conducted latent class trajectory analyses using longitudinal data from the Chinese Longitudinal Healthy Longevity Survey (CLHLS). The objective was to investigate the relationship between long-term dietary diversity development trajectories and frailty to identify potential optimal times for intervention and strategies for mitigating frailty in older adults.

## 2. Methods

### 2.1. Study Design and Participants

A prospective cohort study was utilized to construct a trajectory of dietary diversity and explore its association with frailty using data from CLHLS. The CLHLS is a comprehensive nationwide study conducted with randomly selected samples from half of the counties and cities distributed across 22 out of the 31 provinces in China. This extensive survey encompassed approximately 85% of the entire Chinese population. The questionnaires employed in this study were categorized into two distinct types: one designed for surviving respondents and the other for the families of deceased elderly individuals. The data quality of the CLHLS has been systematically evaluated [22]. Prior to their participation in the study, all subjects provided informed consent for inclusion. Additionally, the study received approval from the Biomedical Ethics Committee of Peking University (IRB00001052-13074).

**Figure 1:**
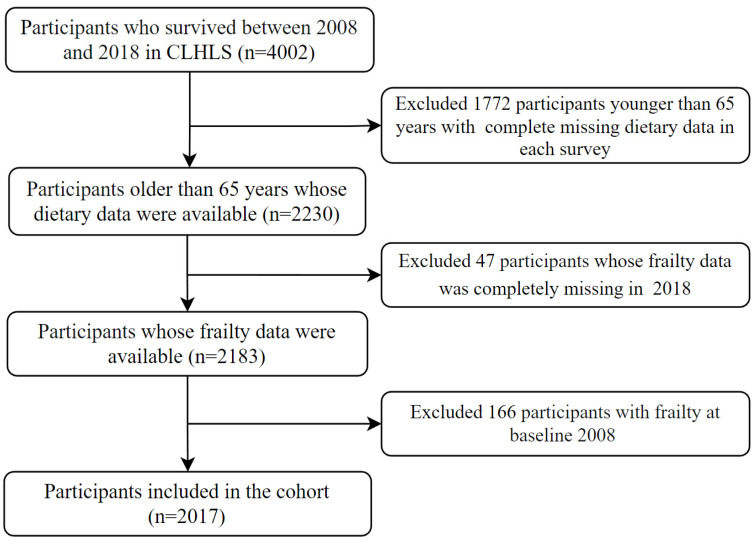
Flow chart for inclusion and exclusion of research subjects.

In this study, individuals who were followed in all four survey waves (2008, 2011, 2014, and 2018) within the CLHLS were included in the cohort. After exclusion, a total of 2017 subjects were ultimately enrolled in this study (Figure 1).

### 2.2. Frailty Index

The frailty index (FI) was constructed following established protocols [23]. Inclusion of deficits associated with health status was contingent upon meeting specific criteria, which included the following: the deficit affecting multiple body systems and spanning various physiological areas; a tendency for the deficit’s prevalence to rise with age; and the deficit not being nearly universal in middle-aged individuals.

The FI was defined as an unweighted count, representing the number of deficits divided by the total possible deficits for an individual. After reviewing self-reported or measured data, a total of 37 indicators encompassing various aspects of self-reported health status, cognitive function, depression, and various chronic diseases were identified in the 2008 and 2018 CLHLSs (Table S1). The components of FI closely resembled those employed in previous research [4,24,25,26]. Each individual item was dichotomized, with a code of one indicating the presence of a deficit. Furthermore, in line with prior studies [27], a score of two was assigned if the respondent had a serious illness that led to hospitalization or confinement to bed on two or more occasions. The FI was subsequently computed by summing all deficits and dividing the sum by the total number of possible deficits, resulting in a range of scores from zero to one. Then, participant classification into non-frail (FI ≤ 0.25) and frail (FI > 0.25) categories was determined based on previously established cutoff points [28].

### 2.3. Dietary Variety Assessment

This study applied the dietary variety score (DVS) to evaluate the initial dietary diversity in the 2008, 2011, 2014, and 2018 CLHLS surveys [18,29]. The intake frequency was used to indicate the intake status of food groups, including fresh fruit, vegetables, meat, fish, eggs, bean products, salted vegetables, sugar, garlic, milk products, nut products, mushrooms or algae, and tea. The DVS was calculated according to the intake frequency of 13 food groups. The specific intake frequency and scoring criteria are shown in Table S2. The total DVS was the sum of the scores of the 13 food groups, with the lowest score being zero and the highest score being 13. The higher the score, the better the dietary diversity.

### 2.4. Covariates

Data for sociodemographic characteristics (e.g., age, sex, ethnicity, illiteracy, economic status, marital status, and co-residence), childhood life status (e.g., place of birth, only child, and hungry in childhood), and lifestyle factors (e.g., smoking, drinking, and physical activity) were collected by trained staff using a questionnaire. More detailed information on the data and measurements collected is available in previous research. The covariates were obtained from the baseline questionnaire [22,30].

### 2.5. Missing Data

To address partial missing data in frailty, diet, and covariates, a multiple imputation approach utilizing chained equations was employed. This approach resulted in the generation of five imputed datasets. The model coefficients were independently estimated within each of these imputed datasets. Subsequently, these estimated coefficients were combined across the imputed datasets using Rubin’s rules [31].

### 2.6. Statistical Analysis

The examination of DVS was conducted at four distinct time points (2008, 2011, 2014, and 2018) using LCTM. The objective was to identify distinct subgroups of individuals whose DVS measurements exhibited similar patterns of change over time. Briefly, this method was designed to identify clusters of individuals following a similar developmental trajectory based on a semiparametric group-based approach. In this study, age, ranging from over 65 to 115, was employed as the timescale for the trajectories. Models based on two to five trajectories were examined, and the optimal model was determined using the Bayesian Information Criterion (BIC). A good model was defined as one with an average posterior probability (APP) exceeding 70% and with at least 5% of individuals belonging to each trajectory group. Three logistic regression models were used to investigate the association between the trajectory group and frailty, and the odds ratio (OR) and 95% confidence interval (CI) were computed. Model 1 was adjusted for sociodemographic characteristics including age, sex, ethnicity, illiteracy, economic status, marital status, and co-residence. Model 2 was additionally adjusted for lifestyle factors, including smoking, drinking, and physical activity. Model 3 was additionally adjusted for childhood life status, including place of birth, only child, and hungry in childhood.

The logistic regression models mentioned above were conducted within various strata defined by age groups (under 75 or 75 years and older), gender, ethnicity, literacy status, economic status, marital status, co-residence, smoking habits, alcohol consumption, exercise habits, place of birth, only-child status, and experiences of childhood hunger. Secondly, likelihood ratio tests were performed to investigate potential statistical interactions between DVS trajectories and place of birth, DVS trajectories and experiences of childhood hunger, as well as DVS trajectories and only-child status. Lastly, for the sensitivity analysis, after eliminating the DVS of zero and one in the baseline survey, latent class trajectory analyses and a logistic regression analysis were performed. Then, people over 100 years old in the 2018 survey were excluded. Moreover, the E-value was estimated to examine the magnitude of an unmeasured confounding factor that could affect the association between dietary diversity development trajectories and frailty by random chance [32].

The LCTM was constructed using the “lcmm” package in the R (version 4.1.0) software. Statistical analyses were performed using SAS (version 9.4), and all tests were two-sided with *p* < 0.05 indicating statistical significance.

## 3. Result

### 3.1. Estimated DVS Trajectory Modeling

**Figure 2:**
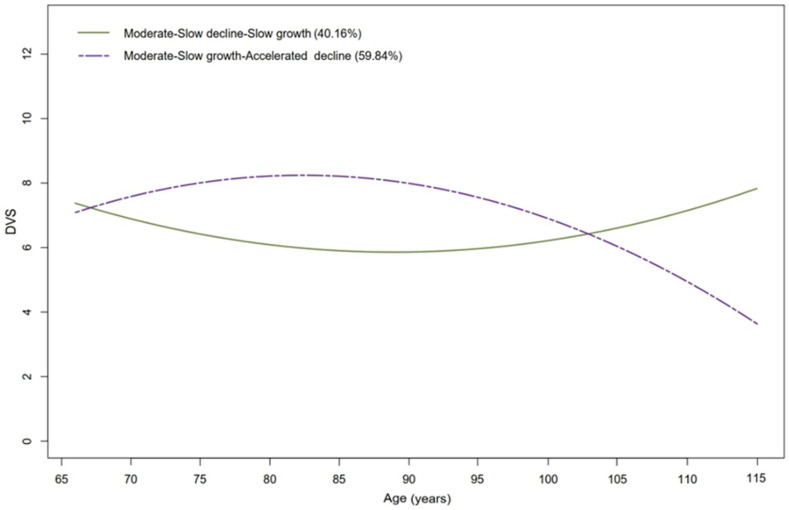
Trajectories of DVS.

Four distinct types of DVS trajectories were explored using LCTM to account for the heterogeneity in participants’ DVS, as detailed in Table S3. The LCTM model with the lowest BIC value was observed to have two trajectories (BIC = 37,725.6), which was considered the most optimal choice. Figure 2 visually depicts these trajectories, illustrating two distinct DVS classes: Class 1 was labeled “Moderate-Slow decline-Slow growth”, comprising 810 (40.16%) individuals; Class 2 was identified as “Moderate-Slow growth-Accelerated decline”, including 1207 (59.84%) individuals.

### 3.2. Baseline Characteristics of Trajectory Subpopulation

The baseline characteristics of participants according to DVS trajectories are shown in Table 1. Notably, individuals belonging to the “Moderate-Slow decline-Slow growth” trajectory group exhibited a higher likelihood of being female, born in rural areas, experiencing childhood hunger, not possessing literacy skills, having a lower economic status, being widowed and never married, residing alone, and not engaging in smoking, alcohol consumption, or regular exercise, compared to those in the “Moderate-Slow growth-Accelerated decline” trajectory group.

### 3.3. Association of DVS Trajectories with Frailty

In this study, the overall prevalence of frailty was 36% (Table S4). Table 2 presents the OR for DVS in the “Moderate-Slow decline-Slow growth” group compared to the “Moderate-Slow growth-Accelerated decline” group. After adjusting for sociodemographic characteristics (Model 1), the OR for DVS in the “Moderate-Slow decline-Slow growth” group was 1.296 (95% CI: 1.054–1.594). Upon further adjustment for lifestyle factors (Model 2), the association between DVS and frailty remained statistically significant; the OR for DVS in the “Moderate-Slow decline-Slow growth” group was 1.323 (95% CI: 1.073–1.632). Furthermore, even after additional adjustment for childhood life status (Model 3), DVS continued to exhibit a significant association with frailty; the OR for DVS in the “Moderate-Slow decline-Slow growth” group was 1.326 (95% CI: 1.075–1.636). Additionally, in the “Moderate-Slow decline-Slow growth” group, it was worth noting that the trajectory of DVS continued to decrease from the early stages of aging, and at 85 to 95 years, it was maintained at a low level.

### 3.4. Subgroup Analysis

**Figure 3:**
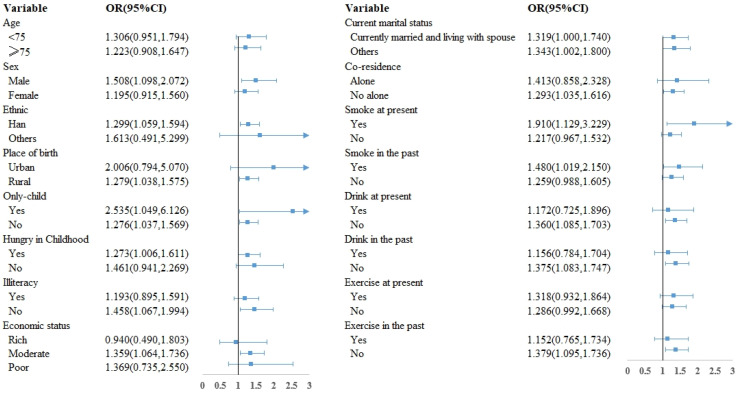
Subgroup analysis. Arrows indicate that the upper or lower limit of the 95% CI of the OR exceeds the horizontal boundary.

Figure 3 displays the outcomes of subgroup analyses. These findings indicated a statistically significant relationship between the “Moderate-Slow decline-Slow growth” trajectories and frailty among specific subgroups including individuals who were male, of Han ethnicity, born in rural areas, experienced childhood hunger, possessed literacy skills, had a moderate economic status, lived with others, currently smoked, had a history of smoking, did not currently consume alcohol, had a history of not drinking alcohol, and did not engage in exercise in the past. Furthermore, this relationship remained unchanged regardless of whether one was an only child or their current marital status.

### 3.5. Interaction Analysis

Moreover, interaction analysis indicated that there was no interaction between the DVS trajectory and any of the three childhood life status variables (place of birth: *P*-interaction = 0.389, only child: *P*-interaction = 0.338, hungry in childhood: *P*-interaction = 0.650).

### 3.6. Sensitivity Analysis

After eliminating the DVS of zero and one in the baseline survey and excluding persons older than 100 years in the 2018 survey, the change trend of the two groups of trajectories (Figures S1 and S2) and the correlation analysis results (Tables S5 and S6) were still consistent with the original analysis results. The E-values of Model 1, Model 2, and Model 3 were 1.54 (confidence interval, 1.19), 1.57 (confidence interval, 1.23), and 1.57 (confidence interval, 1.23), respectively.

## 4. Discussion

In this study, data from the CLHLS cohort spanning from 2008 to 2018 were utilized to evaluate the influence of long-term dietary diversity trajectories on frailty among older Chinese adults. The analysis identified two distinct trajectories of dietary diversity, and a heightened risk of frailty was observed within the group labeled “Moderate-Slow decline-Slow growth” compared to the group labeled “Moderate-Slow growth-Accelerated decline”. Furthermore, to prevent frailty in older adults, it is advisable to implement interventions before 65 years old, with a focus on increasing dietary diversity. These new findings hold significant clinical and public health implications for the prevention and intervention of frailty.

In this study, results showed that the prevalence of frailty was as high as 36%. The prevalence of frailty among Chinese older community dwellers was 10.1% in a previous study [33]. Even after standardizing the two datasets for age and sex, the prevalence of frailty in the study (age: 28.87%, sex: 36.62%) remained higher than what has been reported in previous studies (age: 9.12%, sex: 9.98%). The notable difference was attributed to two primary factors. Firstly, the utilization of the FI encompassed an extensive list of clinical conditions and diseases rather than relying on a few specific signs or symptoms. It drew from a wealth of information, potentially rendering it more sensitive and effective in identifying frailty [34]. Previous studies had suggested that frailty prevalence, as assessed using the FI, tended to be higher than when using the frailty phenotype [2]. Secondly, this study encompassed a larger proportion of older individuals from rural areas. In China, research in underdeveloped or rural regions is relatively limited. Given that frailty tended to be more prevalent in these less developed areas, it was possible that the overall frailty prevalence in China was underestimated in previous studies [33]. Hence, this study provided a more comprehensive representation of frailty prevalence among older people in China. Additionally, the findings of this study indicated that frailty was indeed a more severe and prevalent concern among older individuals in China. This underscored the importance of addressing and managing frailty as a significant public health issue in China.

Indeed, previous studies have investigated the relationship between dietary diversity and frailty. A study of Chinese older adults, also based on CLHLS, found that a more diverse diet was associated with a lower risk of frailty [19], which was consistent with the findings of the present study. However, it measured diet at a single time, ignoring the long-term effects of dietary diversity patterns. This oversight may result in biased estimates when assessing exposure–outcome relationships. For example, based on the identification of two dietary diversity trajectories in this study, the dynamic change trends were different before and after the intersection point. Better than measuring dietary profiles at a single time point, establishing dietary diversity trajectories based on dietary profiles measured over a longer time period may capture the process of dietary dynamics and heterogeneity and, expectedly, identify precisely different populations with varying risks of frailty. Compared to previous studies, the current study demonstrated the feasibility of estimating trends in dietary diversity over a long period of time in the Chinese population. Moreover, the timing of early intervention can be more accurately pinpointed. In the “Moderate-Slow decline-Slow growth” group, although people’s DVS was moderate at age over 65, it has already entered a phase of decline, and the resulting risk of frailty began to increase. Although DVS began to rise after age 85, people were already in the later stages of aging, and the benefits of rising DVS were very limited. Therefore, it was suggested that promoting increased dietary diversity intake in the population in the early stages of aging may be a more effective strategy for preventing the onset of frailty [35].

Since diet represents a long-term cumulative process, it was essential to explore the impact of sustained long-term dietary changes or habits on frailty. The findings suggested that maintaining a high level of dietary diversity was advantageous for preventing frailty, especially in the older population. It may have the following explanations: Firstly, a wider variety of food consumption was associated with increased diversity in the gut microbiota among older individuals [36]. Gut dysbiosis, or an imbalance in the gut microbiota, can trigger an innate immune response and persistent low-grade inflammation [37]. This inflammation was linked to a range of age-related degenerative diseases, including frailty. Secondly, a diverse diet provided a broader spectrum of essential nutrients, ensuring more comprehensive nutritional intake for the body [38]. This enhanced nutritional adequacy was beneficial for preventing frailty in the elderly. Additionally, many nutrients in the body function in coordination with others [39]. The presence and balance of various nutrients influenced the roles of other nutrients. Only when dietary diversity is rich, can multiple nutrients achieve a balanced state, allowing for more effective interactions between nutrients. For instance, the intake of vitamin D, vitamin C, and vitamin K2 can enhance the body’s absorption of calcium, contributing to the prevention of frailty [40,41,42].

In this study, a series of analyses were conducted to assess the reliability of the findings thoroughly. Firstly, consistent conclusions with the main analysis were observed when examining subgroups based on multiple characteristics. Secondly, previous research had consistently demonstrated that variables related to one’s childhood were significantly associated with both phenotypic and functional aging [43]. However, in a previous study, no interaction was found between childhood status and DVS trajectory. The potential impact of extreme values of DVS in the baseline survey and people of older age on the results was also taken into account. Lastly, an innovative sensitivity analysis was introduced using the E-value [32]. When the association between confounders and exposure and outcome reached 1.57 (confidence interval, 1.23), the association between DVS trajectory and frailty in this study appeared to be invalid. Given that 1.57 represents a relatively high association strength, the results can be considered robust.

This study had several potential limitations. First, dietary information was self-reported, which may lead to recall bias. Second, CLHLS did not directly measure dietary intake, and dietary frequency was not exactly equal to dietary intake. Measurement errors in dietary intake assessments were a challenge in epidemiological research, despite ongoing efforts to improve methods. These errors were unlikely to be entirely eliminated due to factors like day-to-day variation and self-reporting limitations [44,45]. In addition, although the E-value was used to assess the effect of unknown confounding factors on the results, the possibility of residual confounding cannot be excluded due to the observational nature of the studies. Finally, this study focused on the effect of dietary diversity on frailty without considering people’s different dietary preferences, even though their DVS may be the same. In the upcoming research, the inclusion of dietary intake is planned to provide a more comprehensive exploration of the relationship between dietary factors and frailty.

## 5. Conclusions

In conclusion, the latent class trajectory analysis revealed that in Chinese older adults, individuals with a DVS change trajectory characterized by “Moderate-Slow decline-Slow growth” were at an increased risk of frailty. The optimal time for intervention is in the early stages of aging. These novel findings had significant clinical and public health implications.

## Figures and Tables

**Table 1:** Baseline characteristics of participants based on the trajectories of DVS.

Variables	Overall *n* = 2017	Moderate-Slow Decline-Slow Growth *n* = 810	Moderate-Slow Growth-Accelerated Decline *n* = 1207	*p*-Value ^a^
Age, M (P25, P75)	74 (69, 80)	75 (70, 80)	73 (69, 80)	<0.001
Sex, *n* (%)				
Male	969 (48.04)	317 (39.14)	652 (54.02)	<0.001
Female	1048 (51.96)	493 (60.86)	555 (45.98)	
Ethnicity, *n* (%)				
Han	1894 (93.9)	740 (91.36)	1154 (95.61)	<0.001
Hui	5 (0.25)	1 (0.12)	4 (0.33)	
Zhuang	74 (3.67)	47 (5.8)	27 (2.24)	
Yao	11 (0.55)	5 (0.62)	6 (0.5)	
Man	6 (0.3)	2 (0.25)	4 (0.33)	
Others	27 (1.34)	15 (1.85)	12 (0.99)	
Place of birth, *n* (%)				
Urban	155 (7.68)	42 (5.19)	113 (9.36)	<0.001
Rural	1860 (92.22)	767 (94.69)	1093 (90.56)	
Missing	2 (0.1)	1 (0.12)	1 (0.08)	
Only child, *n* (%)				
Yes	150 (7.44)	61 (7.53)	89 (7.37)	0.895
No	1867 (92.56)	749 (92.47)	1118 (92.63)	
Hungry in Childhood, *n* (%)				
Yes	1454 (72.09)	614 (75.8)	840 (69.59)	<0.001
No	515 (25.53)	171 (21.11)	344 (28.5)	
Missing	48 (2.38)	25 (3.09)	23 (1.91)	
Illiteracy, *n* (%)				
Yes	991 (49.13)	478 (59.01)	513 (42.5)	<0.001
No	1026 (50.87)	332 (40.99)	694 (57.5)	
Economic status, *n* (%)				
Very rich	22 (1.09)	5 (0.62)	17 (1.41)	<0.001
Rich	217 (10.76)	54 (6.67)	163 (13.5)	
Moderate	1469 (72.83)	581 (71.73)	888 (73.57)	
Poor	270 (13.39)	142 (17.53)	128 (10.6)	
Very poor	37 (1.83)	27 (3.33)	10 (0.83)	
Missing	2 (0.1)	1 (0.12)	1 (0.08)	
Current marital status, *n* (%)				
Married and living with spouse	1172 (58.11)	411 (50.74)	761 (63.05)	<0.001
Separated	61 (3.02)	26 (3.21)	35 (2.9)	
Divorced	5 (0.25)	2 (0.25)	3 (0.25)	
Widowed	758 (37.58)	361 (44.57)	397 (32.89)	
Never married	21 (1.04)	10 (1.23)	11 (0.91)	
Co-residence, *n* (%)				
With household member	1673 (82.94)	638 (78.77)	1035 (85.75)	<0.001
Alone	330 (16.36)	169 (20.86)	161 (13.34)	
In an institution	14 (0.69)	3 (0.37)	11 (0.91)	
Smoke at present, *n* (%)				
Yes	470 (23.3)	174 (21.48)	296 (24.52)	0.113
No	1547 (76.7)	636 (78.52)	911 (75.48)	
Smoke in the past, *n* (%)				
Yes	699 (34.66)	227 (28.02)	472 (39.11)	<0.001
No	1314 (65.15)	581 (71.73)	733 (60.73)	
Missing	4 (0.2)	2 (0.25)	2 (0.17)	
Drink at present, *n* (%)				
Yes	455 (22.56)	155 (19.14)	300 (24.86)	0.003
No	1562 (77.44)	655 (80.86)	907 (75.14)	
Drink in the past, *n* (%)				
Yes	621 (30.79)	203 (25.06)	418 (34.63)	<0.001
No	1392 (69.01)	605 (74.69)	787 (65.2)	
Missing	4 (0.2)	2 (0.25)	2 (0.17)	
Exercise at present, *n* (%)				
Yes	732 (36.29)	237 (29.26)	495 (41.01)	<0.001
No	1285 (63.71)	573 (70.74)	712 (58.99)	
Exercise in the past, *n* (%)				
Yes	556 (27.57)	183 (22.59)	373 (30.9)	<0.001
No	1455 (72.14)	624 (77.04)	831 (68.85)	
Missing	6 (0.3)	3 (0.37)	3 (0.25)	

^a^ Based on a Wilcoxon signed-rank test or Chi-square statistics as appropriate.

**Table 2:** Logistic regression models for frailty and DVS trajectories.

	Moderate-Slow Decline-Slow Growth	Moderate-Slow Growth-Accelerated Decline
Subjects, *n*	810	1207
Frailty cases, *n*	334	393
OR (95% CI)		
Model 1	1.296 (1.054–1.594)	Reference
Model 2	1.323 (1.073–1.632)	Reference
Model 3	1.326 (1.075–1.636)	Reference

Model 1 was adjusted for age, sex, ethnicity, illiteracy, economic status, marital status, and co-residence; Model 2 was adjusted for Model 1 + smoking, drinking, and physical activity; Model 3 was adjusted for Model 2 + place of birth, only child, and hungry in childhood; OR, odds ratio; CI, confidence interval.

## Data Availability

The data of CLHLS are available at https://opendata.pku.edu.cn/dataverse/CHADS (accessed on 15 March 2023).

## Supplementary Materials

The following supporting information can be downloaded at https://www.mdpi.com/article/10.3390/nu16101445/s1, Table S1: List of items included in the frailty index; Table S2: Food intake frequency and scoring criteria; Table S3: Fitting statistics for DVS trajectories; Table S4: The prevalence of frailty; Figure S1: Trajectories of DVS after eliminating the lowest 2% DVS in the baseline survey; Table S5: Logistic regression models for frailty and DVS trajectories after eliminating the lowest 2% DVS in the baseline survey; Figure S2: Trajectories of DVS after excluding persons older than 100 years in the 2018 survey; Table S6: Logistic regression models for frailty and DVS trajectories after excluding persons older than 100 years in the 2018 survey.

## References

[1] Hoogendijk E.O., Afilalo J., Ensrud K.E., Kowal P., Onder G., Fried L.P. (2019). Frailty: Implications for clinical practice and public health. Lancet.

[2] O’Caoimh R., Sezgin D., O’Donovan M.R., Molloy D.W., Clegg A., Rockwood K., Liew A. (2021). Prevalence of frailty in 62 countries across the world: A systematic review and meta-analysis of population-level studies. Age Ageing.

[3] Ida S., Kaneko R., Imataka K., Murata K. (2019). Relationship between frailty and mortality, hospitalization, and cardiovascular diseases in diabetes: A systematic review and meta-analysis. Cardiovasc. Diabetol..

[4] Fan J., Yu C., Guo Y., Bian Z., Sun Z., Yang L., Chen Y., Du H., Li Z., Lei Y. (2020). Frailty index and all-cause and cause-specific mortality in Chinese adults: A prospective cohort study. Lancet Public Health.

[5] Angulo J., El Assar M., Rodriguez-Manas L. (2016). Frailty and sarcopenia as the basis for the phenotypic manifestation of chronic diseases in older adults. Mol. Aspects Med..

[6] Arauna D., Cerda A., Garcia-Garcia J.F., Wehinger S., Castro F., Mendez D., Alarcon M., Fuentes E., Palomo I. (2020). Polypharmacy Is Associated with Frailty, Nutritional Risk and Chronic Disease in Chilean Older Adults: Remarks from PIEI-ES Study. Clin. Interv. Aging.

[7] Liperoti R., Vetrano D.L., Palmer K., Targowski T., Cipriani M.C., Lo Monaco M.R., Giovannini S., Acampora N., Villani E.R., Bernabei R. (2021). Association between frailty and ischemic heart disease: A systematic review and meta-analysis. BMC Geriatr..

[8] Dent E., Martin F.C., Bergman H., Woo J., Romero-Ortuno R., Walston J.D. (2019). Management of frailty: Opportunities, challenges, and future directions. Lancet.

[9] Gale C.R., Westbury L., Cooper C. (2018). Social isolation and loneliness as risk factors for the progression of frailty: The English Longitudinal Study of Ageing. Age Ageing.

[10] Kehler D.S., Theou O. (2019). The impact of physical activity and sedentary behaviors on frailty levels. Mech. Ageing Dev..

[11] Yang G., Cao X., Li X., Zhang J., Ma C., Zhang N., Lu Q., Crimmins E.M., Gill T.M., Chen X. (2022). Association of Unhealthy Lifestyle and Childhood Adversity With Acceleration of Aging Among UK Biobank Participants. JAMA Netw. Open.

[12] Otaki N., Yano M., Yokoro M., Tanino N., Fukuo K., Raymo J. (2021). Relationship Between Dietary Variety and Frailty in Older Japanese Women During the Period of Restriction on Outings due to COVID-19. J. Gerontol. Ser. B.

[13] Hayakawa M., Motokawa K., Mikami Y., Yamamoto K., Shirobe M., Edahiro A., Iwasaki M., Ohara Y., Watanabe Y., Kawai H. (2021). Low Dietary Variety and Diabetes Mellitus Are Associated with Frailty among Community-Dwelling Older Japanese Adults: A Cross-Sectional Study. Nutrients.

[14] Kiuchi Y., Makizako H., Nakai Y., Tomioka K., Taniguchi Y., Kimura M., Kanouchi H., Takenaka T., Kubozono T., Ohishi M. (2021). The Association between Dietary Variety and Physical Frailty in Community-Dwelling Older Adults. Healthcare.

[15] Yokoro M., Otaki N., Yano M., Tani M., Tanino N., Fukuo K. (2023). Associations between Dietary Variety and Frailty in Community-Dwelling Older People Who Live Alone: Gender Differences. J. Frailty Aging.

[16] Yokoro M., Otaki N., Yano M., Imamura T., Tanino N., Fukuo K. (2023). Low Dietary Variety Is Associated with Incident Frailty in Older Adults during the Coronavirus Disease 2019 Pandemic: A Prospective Cohort Study in Japan. Nutrients.

[17] Xu X., Inglis S.C., Parker D. (2021). Sex differences in dietary consumption and its association with frailty among middle-aged and older Australians: A 10-year longitudinal survey. BMC Geriatr..

[18] Aihemaitijiang S., Zhang L., Ye C., Halimulati M., Huang X., Wang R., Zhang Z. (2022). Long-Term High Dietary Diversity Maintains Good Physical Function in Chinese Elderly: A Cohort Study Based on CLHLS from 2011 to 2018. Nutrients.

[19] Wang X.-M., Zhong W.-F., Li Z.-H., Chen P.-L., Zhang Y.-J., Ren J.-J., Liu D., Shen Q.-Q., Yang P., Song W.-Q. (2023). Dietary diversity and frailty among older Chinese people: Evidence from the Chinese Longitudinal Healthy Longevity Study. Am. J. Clin. Nutr..

[20] Lennon H., Kelly S., Sperrin M., Buchan I., Cross A.J., Leitzmann M., Cook M.B., Renehan A.G. (2018). Framework to construct and interpret latent class trajectory modelling. BMJ Open.

[21] Lu L., Contrand B., Dupuy M., Ramiz L., Sztal-Kutas C., Lagarde E. (2022). Mental and physical health among the French population before and during the first and second COVID-19 lockdowns: Latent class trajectory analyses using longitudinal data. J. Affect. Disord..

[22] Zeng Y., Feng Q., Hesketh T., Christensen K., Vaupel J.W. (2017). Survival, disabilities in activities of daily living, and physical and cognitive functioning among the oldest-old in China: A cohort study. Lancet.

[23] Searle S.D., Mitnitski A., Gahbauer E.A., Gill T.M., Rockwood K. (2008). A standard procedure for creating a frailty index. BMC Geriatr..

[24] Gu D., Dupre M.E., Sautter J., Zhu H., Liu Y., Yi Z. (2009). Frailty and Mortality Among Chinese at Advanced Ages. J. Gerontol. Ser. B Psychol. Sci. Soc. Sci..

[25] Kulminski A., Yashin A., Ukraintseva S., Akushevich I., Arbeev K., Land K., Manton K. (2006). Accumulation of health disorders as a systemic measure of aging: Findings from the NLTCS data. Mech. Ageing Dev..

[26] Mitnitski A., Song X., Skoog I., Broe G.A., Cox J.L., Grunfeld E., Rockwood K. (2005). Relative fitness and frailty of elderly men and women in developed countries and their relationship with mortality. J. Am. Geriatr. Soc..

[27] Goggins W.B., Woo J., Sham A., Ho S.C. (2005). Frailty index as a measure of biological age in a Chinese population. J. Gerontol. A Biol. Sci. Med. Sci..

[28] Song Y., Deng Y., Li J., Hao B., Cai Y., Chen J., Shi H., Xu W. (2021). Associations of falls and severe falls with blood pressure and frailty among Chinese community-dwelling oldest olds: The Chinese Longitudinal Health and Longevity Study. Aging.

[29] Zhang J., Wang Q., Hao W., Zhu D. (2022). Long-Term Food Variety and Dietary Patterns Are Associated with Frailty among Chinese Older Adults: A Cohort Study Based on CLHLS from 2014 to 2018. Nutrients.

[30] Lv Y.-B., Gao X., Yin Z.-X., Chen H.-S., Luo J.-S., Brasher M.S., Kraus V.B., Li T.-T., Zeng Y., Shi X.-M. (2018). Revisiting the association of blood pressure with mortality in oldest old people in China: Community based, longitudinal prospective study. BMJ.

[31] Rubin D.B. (1987). Multiple Imputation for Nonresponse in Surveys.

[32] VanderWeele T.J., Ding P. (2017). Sensitivity Analysis in Observational Research: Introducing the E-Value. Ann. Intern. Med..

[33] Zhou Q., Li Y., Gao Q., Yuan H., Sun L., Xi H., Wu W. (2023). Prevalence of Frailty among Chinese Community-Dwelling Older Adults: A Systematic Review and Meta-Analysis. Int. J. Public Health.

[34] Cesari M., Gambassi G., van Kan G.A., Vellas B. (2014). The frailty phenotype and the frailty index: Different instruments for different purposes. Age Ageing.

[35] Colizzi M., Lasalvia A., Ruggeri M. (2020). Prevention and early intervention in youth mental health: Is it time for a multidisciplinary and trans-diagnostic model for care?. Int. J. Ment. Health Syst..

[36] Claesson M.J., Jeffery I.B., Conde S., Power S.E., O’Connor E.M., Cusack S., Harris H.M.B., Coakley M., Lakshminarayanan B., O’Sullivan O. (2012). Gut microbiota composition correlates with diet and health in the elderly. Nature.

[37] Tiihonen K., Ouwehand A.C., Rautonen N. (2010). Human intestinal microbiota and healthy ageing. Ageing Res. Rev..

[38] Schuette L.K., Song W.O., Hoerr S.L. (1996). Quantitative use of the Food Guide Pyramid to evaluate dietary intake of college students. J. Am. Diet. Assoc..

[39] Mirmiran P., Azadbakht L., Esmaillzadeh A., Azizi F. (2004). Dietary diversity score in adolescents—A good indicator of the nutritional adequacy of diets: Tehran lipid and glucose study. Asia Pac. J. Clin. Nutr..

[40] DeLuca H.F. (2004). Overview of general physiologic features and functions of vitamin D. Am. J. Clin. Nutr..

[41] Morcos S.R., El-Shobaki F.A., El-Hawary Z., Saleh N. (1976). Effect of vitamin C and carotene on the absorption of calcium from the intestine. Z. Ernährungswissenschaft.

[42] Gasmi A., Bjørklund G., Peana M., Mujawdiya P.K., Pivina L., Ongenae A., Piscopo S., Severin B. (2022). Phosphocalcic metabolism and the role of vitamin D, vitamin K2, and nattokinase supplementation. Crit. Rev. Food Sci. Nutr..

[43] Cao X., Ma C., Zheng Z., He L., Hao M., Chen X., Crimmins E.M., Gill T.M., Levine M.E., Liu Z. (2022). Contribution of life course circumstances to the acceleration of phenotypic and functional aging: A retrospective study. eClinicalMedicine.

[44] Tarasuk V.S., Brooker A.S. (1997). Interpreting epidemiologic studies of diet-disease relationships. J. Nutr..

[45] Satija A., Yu E., Willett W.C., Hu F.B. (2015). Understanding nutritional epidemiology and its role in policy. Adv. Nutr..

