# Supplementary material for: Associations of Dietary Diversity Trajectories with Frailty among Chinese Older Adults: A Latent Class Trajectory Analysis Based on a CLHLS Cohort
Source: Nutrients. 2024 May 10;16(10):1445. doi: 10.3390/nu16101445 (PMC11124478; doi:10.3390/nu16101445)
Supplement: Supplementary file 1 [file nutrients-16-01445-s001.zip › nutrients-2966031-supplementary.pdf]

Table S1: List of items included in the frailty index

No	Items	Coding of variables
1	Needs assistance bathing	Yes=1.00; no=0.00
2	Needs assistance dressing	Yes=1.00; no=0.00
3	Needs assistance toileting	Yes=1.00; no=0.00
4	Needs assistance in indoor transferring	Yes=1.00; no=0.00
5	Needs assistance eating	Yes=1.00; no=0.00
6	Incontinence	Yes=1.00; no=0.00
7	Unable to visit neighbors by oneself	Yes=1.00; no=0.00
8	Unable to shop by oneself if necessary	Yes=1.00; no=0.00
9	Unable to cook meals by oneself if necessary	Yes=1.00; no=0.00
10	Unable to wash clothing by oneself	Yes=1.00; no=0.00
11	Unable to walk continuously for 1 kilometer	Yes=1.00; no=0.00
12	Unable to lift a weight of 5 kg	Yes=1.00; no=0.00
13	Unable to continuously crouch and stand up three times	Yes=1.00; no=0.00
14	Unable to use public transportation	Yes=1.00; no=0.00
15	Unable to put hand behind neck	Yes=1.00; no=0.00
16	Unable to put hand behind lower back	Yes=1.00; no=0.00
17	Unable to raise arm upright	Yes=1.00; no=0.00
18	Unable to stand up from sitting in a chair	Yes=1.00; no=0.00
19	Unable to pick up a book from the floor	Yes=1.00; no=0.00
20	Cognitively impaired (based on the Mini Mental State Examination)	<6=1.00;<br>≥6 and <12=0.75;<br>≥12 and <18=0.25;<br>≥18=0.00
21	Poor self-rated health	Yes=1.00; no=0.00
22	Poor interviewer-rated health	Yes=1.00; no=0.00
23	Number of serious illnesses in the past	0=0.00;<br>1=1.00;<br>≥2=2.00

No	Items	Coding of variables
24	Loneliness of psychological distress	Yes=1.00; no=0.00
25	usefulness of psychological distress	Yes=1.00; no=0.00
26	fearfulness of psychological distress	Yes=1.00; no=0.00
27	Suffering from hypertension	Yes=1.00; no=0.00
28	Suffering from diabetes	Yes=1.00; no=0.00
29	Suffering from tuberculosis	Yes=1.00; no=0.00
30	Suffering from heart disease	Yes=1.00; no=0.00
31	Suffering from stroke/cerebrovascular disease	Yes=1.00; no=0.00
32	Suffering from bronchitis, emphysema, asthma, or pneumonia	Yes=1.00; no=0.00
33	Suffering from cancer	Yes=1.00; no=0.00
34	Suffering from arthritis	Yes=1.00; no=0.00
35	Suffering from bedsores	Yes=1.00; no=0.00
36	Suffering from gastric or duodenal ulcers	Yes=1.00; no=0.00
37	Suffering from Parkinson’s disease	Yes=1.00; no=0.00

Table S2: Food intake frequency and scoring criteria

No	Varieties	Coding of variables
1	Fruit	daily or often=1;<br>occasionally or hardly ever=0
2	Vegetable	daily or often=1;<br>occasionally or hardly ever=0
3	Meat	daily or weekly=1;<br>monthly or occasionally or almost never=0
4	Fish	daily or weekly=1;<br>monthly or occasionally or almost never=0
5	Eggs	daily or weekly=1;<br>monthly or occasionally or almost never=0
6	Bean products	daily or weekly=1;<br>monthly or occasionally or almost never=0
7	Salted vegetables	daily or weekly=1;<br>monthly or occasionally or almost never=0
8	Sugar	daily or weekly=1;<br>monthly or occasionally or almost never=0
9	Garlic	daily or weekly=1;<br>monthly or occasionally or almost never=0
10	Milk products	daily or weekly=1;<br>monthly or occasionally or almost never=0
11	Nut products	daily or weekly=1;<br>monthly or occasionally or almost never=0
12	Mushrooms or algae	daily or weekly=1;<br>monthly or occasionally or almost never=0
13	Tea	daily or weekly=1;<br>monthly or occasionally or almost never=0

Table S3: Fitting statistics for DVS trajectories

	Number of trajectory											
	2			3			4			5		
BIC[a]	37725.6			37771.93			37807.95			37832.3		
Class1(n/%/APP[b])	810	40.16	0.716	307	15.22	0.716	83	4.12	0.493	0	0	0
Class2(n/%/APP[b])	1207	59.84	0.743	1417	70.25	0.685	1434	71.10	0.679	601	29.80	0.742
Class3(n/%/APP[b])	/	/	/	293	14.53	0.711	159	7.88	0.493	1323	65.59	0.732
Class4(n/%/APP[b])	/	/	/	/	/	/	341	16.91	0.717	93	4.61	0.643
Class5(n/%/APP[b])	/	/	/	/	/	/	/	/	/	0	0	0

a: The lower absolute value shows a better model fit; b: The higher value is better (> 0.7 in a class), BIC: Bayesian information criteria; APP average posterior probabilities.

Table S4: The prevalence of frailty

Prevalence of frailty (%)	Sex		Total
	Male	Female	
Age (years)			
65-74	17.83	28.70	23.25
≥75	44.24	56.50	50.91
Total	29.41	42.18	36.00

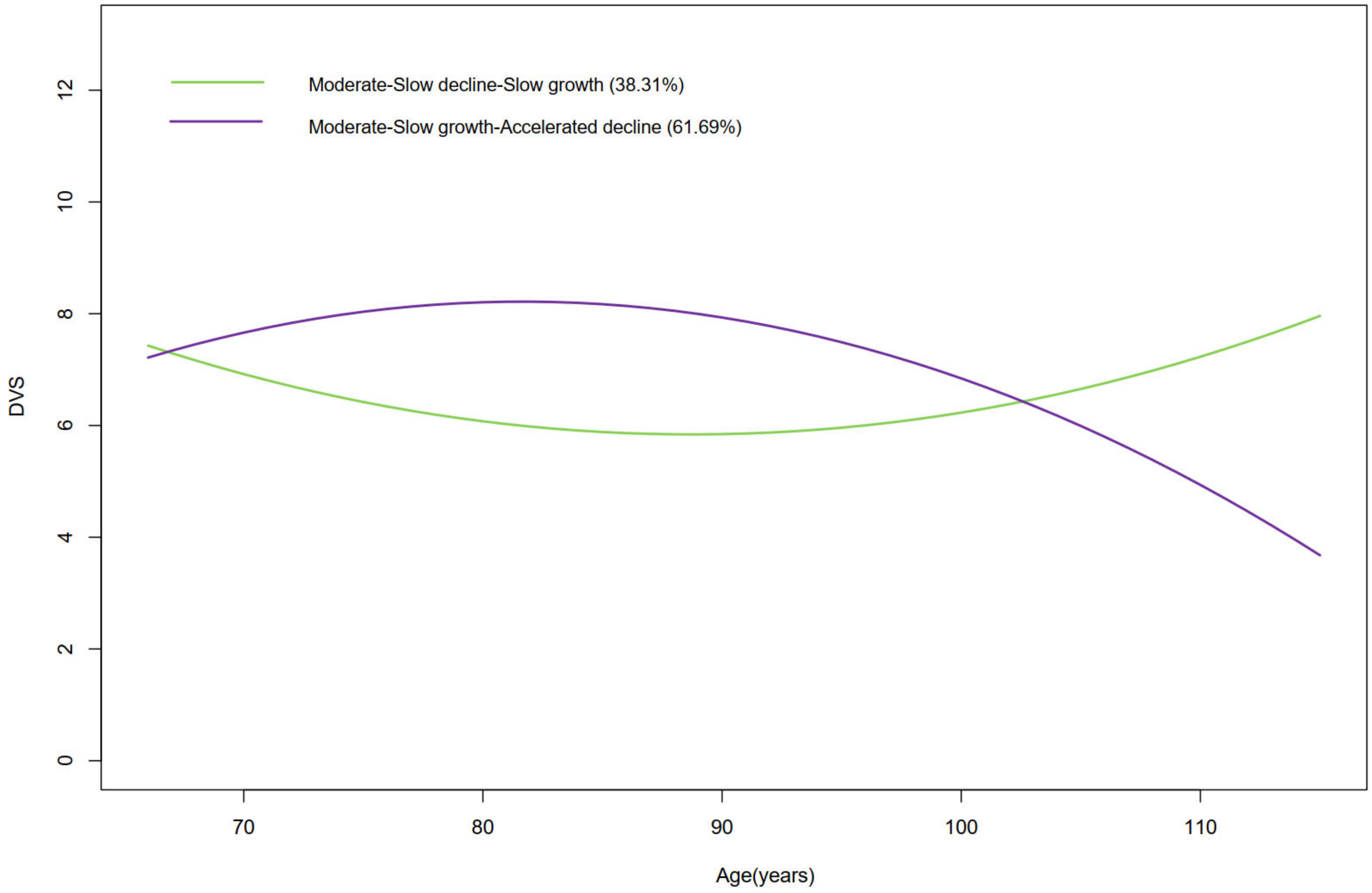

Figure S1: Trajectories of DVS after eliminating the lowest 2% DVS in the baseline survey

Table S5: Logistic regression models for frailty and DVS trajectories after eliminating the lowest 2% DVS in the baseline survey

	**Moderate-Slow decline-Slow growth**	**Moderate-Slow growth-Accelerated decline**
Subjects, n	762	1227
Frailty cases, n	311	401
OR (95%CI)		
Model 1	1.267(1.025,1.565)	Reference
Model 2	1.291(1.042,1.601)	Reference
Model 3	1.296(1.044,1.608)	Reference

Model 1 was adjusted for age, sex, ethnic, illiteracy, economic status, marital status and co-residence; Model 2 was adjusted for Model 2 + smoking, drinking and physical activity; Model 3 was adjusted for Model 2 + place of birth, only-child and hungry in childhood, OR, odds ratio; CI, confidence interval.

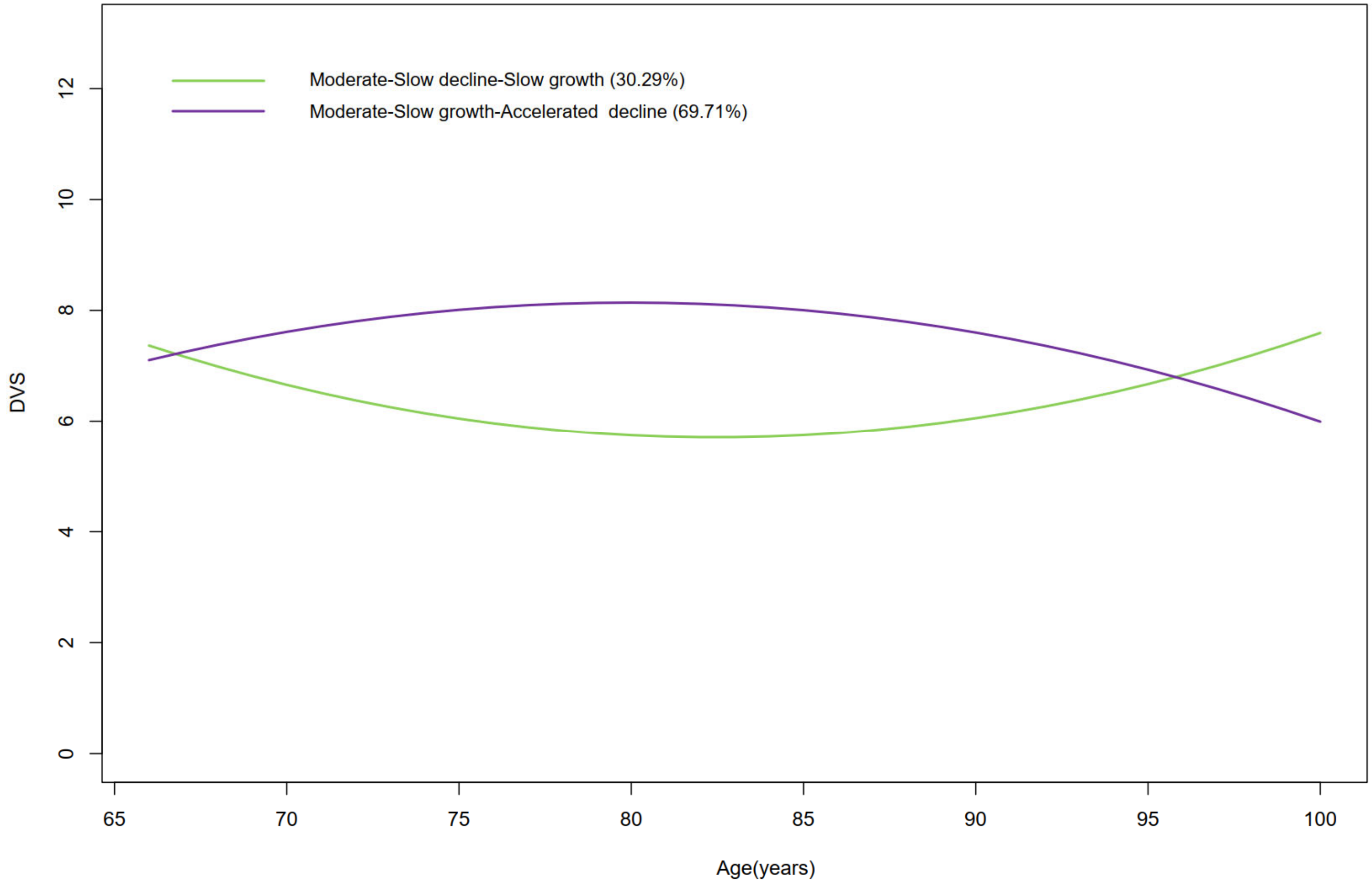

Figure S2: Trajectories of DVS after excluding persons older than 100 years in the 2018 survey

Table S6: Logistic regression models for frailty and DVS trajectories after excluding persons older than 100 years in the 2018 survey

	Moderate-Slow decline-Slow growth	Moderate-Slow growth-Accelerated decline
Subjects, n	590	1358
Frailty cases, n	245	435
OR (95%CI)		
Model 1	1.296(1.033,1.627)	Reference
Model 2	1.320(1.050,1.661)	Reference
Model 3	1.318(1.046,1.660)	Reference

Model 1 was adjusted for age, sex, ethnic, illiteracy, economic status, marital status and co-residence; Model 2 was adjusted for Model 2 + smoking, drinking and physical activity; Model 3 was adjusted for Model 2 + place of birth, only-child and hungry in childhood, OR, odds ratio; CI, confidence interval.
